# Medical and Philosophical Intelligence

**Published:** 1813-11

**Authors:** 


### MEDICAL AND PHILOSOPHICAL INTELLIGENCE.

ROYAL SOCIETY.—On Thursday the 1st of July, a paper by Sir Humphry Davy was read, containing farther observations on the new detonating compound of chlorine and azote. After recovering from the accident which happened to him during his original experiments on this substance, Sir Humphry made a variety of experiments to determine its properties and composition. Its specific gravity is 1·623. When in contact with water, it congeals at about 40°; but this does not happen when it is kept separate from water. It detonates in nitric acid, and in ammonia; gives out azote in muriatic acid; and is likewise decomposed in sulphuric acid. Attempts were made to decompose it in exhausted glass vessels in the state of vapor; but they were unsuccessful. In general, the glass was broken by an explosion; and when that did not happen, the proportion of chlorine and azote evolved could not be determined, on account of the unknown proportion of atmospheric air that remained in the vessel. When the detonating compound is brought in contact with mercury, a white powder is formed, and azotic gas disengaged. In one experiment a detonation took place, which obliged him to work upon smaller quantities. The white powder was found to be a mixture of calomel and corrosive sublimate; and it sublimed entirely without the disengagement of any gas, indicating the absence both of hydrogen and oxygen. Muriatic acid does not destroy the color of solution of indigo in sulphuric acid; but if it be impregnated with chlorine, it destroys a determinate quantity of the blue color, according to the proportion of chlorine present. The same thing happens when the detonating compound is dissolved in muriatic acid. This furnished a method for determining the proportion of chlorine contained in the detonating compound. The result of all these methods of analysis is, that the detonating compound is composed of

| | |
|---|---|
| Chlorine . . . . . . . | 91 |
| Azote . . . . . . . | 9 |
| | 100 |

reckoning by weight, or if we reckon by bulks of

| | |
|---|---|
| Chlorine . . . . . . . | 400 |
| Azotic gas . . . . . . . | 100 |
| | 500 |

Sir H. Davy proposes to call this detonating compound *azotane*.

At the same meeting were read some observations on a new comet, observed by Captain Hill, in the Hon. East India Company's service.

On Thursday, the 8th of July, the following papers were read:

1. A catalogue of the positions of a number of circumpolar stars by the Astronomer Royal, Mr. Pond.

2. An analysis of a substance thrown out of Mount Vesuvius, by

James Smithson, Esq, This substance had been sent to Mr. Smithson when in Italy in 1794, in order to determine its nature; and he ascertained, by a number of trials, that it consisted chiefly of sulphate of potash. This result was published soon after in an Italian Journal, but no subsequent notice was taken of it by mineralogists. Mr. Smithson was induced to examine it with more accuracy lately, and the result of his experiments is, that it consists of sulphate of potash, sulphate of soda, sulphate of ammonia, muriate of ammonia, muriate of copper, and muriate of iron, with some earthy matter. Mr. Smithson, by way of introduction to his paper, gives a view of his opinions about the origin of the earth. In his opinion, it was originally a sun, or a comet, and was brought to the state in which it is at present by undergoing combustion at the surface. The volcanoes are the relics of this original combustion, and the materials were the metallic bases of the earthy substances of which the primitive strata are composed. As a proof that these primitive strata have been formed by combustion, he mentions that garnets, hornblende, and other crystals found in them, contain no water, and that little or no water is to be found in the primitive strata themselves.

3. Observations, by Dr. Marcet, on the cold produced by the evaporation of sulphuret of carbon. This liquid evaporates more rapidly than any other, and produces in consequence a greater degree of cold. A spirit of wine thermometer, having its bulb surrounded with cotton cloth or lint, if dipped into sulphuret of carbon, and suspended in the air, sinks from 60° to 0. If it be put into the receiver of an air-pump, and a moderate exhaustion be made, it sinks from 60° to — 81° (I have seen it myself in these circumstances sink from 74° to — 72°). If a tube containing mercury be treated in the same way, the mercury may be readily frozen, even in summer. The drier the air in the receiver is, the more easily is the cold produced. Hence the presence of sulphuric acid may be of some little service in removing the vapor from the air in the receiver previous to exhaustion; otherwise it occasions no increase of the cold.

4. Observations on the composition of fluor spar, and on its acid basis, by Sir Humphry Davy. The author begins his paper with an historical detail of the attempts made by himself, and Gay-Lussac and Thenard, to decompose fluoric acid, and the ill success of these attempts. It appears from the compounds into which fluoric acid enters, that the weight of an integrant particle of it does not exceed 1·05, supposing an atom of oxygen to weigh 1. Hence it follows that if it is a compound of oxygen and an inflammable base, the base can only have the 20th part of the weight of the oxygen. This supposition he considers as unlikely to be correct. He therefore supposes that fluoric acid, like muriatic acid, is a compound of hydrogen and an unknown supporter of combustion, to which he gives the name of fluorine. He relates many experiments made in order to obtain fluorine in a separate state, but none of them were attended with success. As chlorine has the property of decomposing several oxides, and driving off their oxygen, it occurred to him as likely that it might in certain cases drive off fluorine. Fluate of silver and fluate of mercury, with this view, were acted upon by chlorine. The fluoric acid (or fluorine) was disengaged, and horn silver and corrosive sublimate formed; but no fluorine was set free. When the experiments were made in glass retorts, the glass was corroded, and silicated fluoric acid gas obtained. When they were made in platinum vessels, the metal was corroded, and a red or brown powder formed. It would seem from the trials made that fluorine has so violent an action on all other bodies that it is very difficult, if not impossible, to obtain it in a separate state. The author promises to continue this subject in a subsequent paper.

5. A paper, by Mr. Alman, on the method of freeing equations from surds, though the roots be pretty high in their dimensions. From the nature of this paper it could not be read at full length, so that it is not possible to give any idea of the method employed by the author; but he referred to a method previously given in the Irish Transactions by Mr. Money, which he considers as general, and which, probably, constitutes the foundation of his own.

The Society adjourned till the 4th of November next.

---

*Galvanic Battery.*—On Saturday, the 2d of July, J. G. Children, esq. put in action the greatest galvanic battery that has ever been constructed. It consisted of 20 pair of copper and zinc plates, each plate 6 feet in length, 2 feet 8 inches in breadth. Each pair was fixed together at the top by pieces of lead cut into ribbons. A separate wooden cell was constructed for each pair. The plates were suspended from a wooden beam fixed at the ceiling, and were so hung by means of counterpoises that they could be easily raised or let down into the cells. The cells were filled with water, containing a mixture of sulphuric and nitric acids. At first the acids amounted to 1-60th of the water; but more was gradually added till it amounted to the 30th. Leaden pipes were attached to the two extremities of the battery, and conveyed the electricity out of doors to an adjoining shade, where the experiments were made. The power of this battery was very great; though I am not certain whether it increased in proportion to the size of the plates. It ignited about 6 feet in length of thick platinum wire. The heat produced was very intense. It melted platinum with great facility. Iridium was likewise melted into a globule, and proved to be a brittle metal. The ore of iridium and osmium was likewise melted, but not so completely. Charcoal was kept in a white heat in chlorine gas, and in phosgene gas; but no change took place in either of these gases. Neither tungsten nor uranium underwent any change. A very singular fact was pointed out by the sagacity of Dr. Wollaston, and succeeded upon trial. A greater length of thick platinum wire was ignited than of platinum wire of a much smaller size. This Dr. Wollaston had previously ascertained in his own minute galvanic batteries, consisting of a single pair of small plates.

---

*Volatility of Cerium.*—The volatility of this metal, which had been previously inferred from the experiments of Vauquelin, was fully confirmed in Mr. Children's laboratory at Tonbridge. A quantity tity tity of oxalate of cerium had been prepared in order to obtain from it the oxide of the metal. This oxalate was exposed in a charcoal crucible within a tobacco-pipe mouth to the strongest heat that could be raised in a forge. In this heat the cerium was volatilized so completely that not the least trace of it remained.

---

*Action of the Agate on Light.*—Dr. Brewster has now established, by numerous experiments, that the nebulous light of the agate has the same relation to the bright image as the extraordinarily refracted image has to the ordinarily refracted image of all doubly refracting crystals. There is still, however, no appearance of the nebulous light being produced by a higher refractive power than the bright image. All the phenomena of polarization are produced when the plate of agate is less than the $\frac{1}{150}$th of an inch; and the nebulous light, when in its evanescent state, can be revived by depolarization with mica, in every respect like one of the images formed by double refraction. The colored appearances in the agate, to which mineralogists have given the name of iridescence, furnish a series of the most singular results, arising, apparently, from the mechanical structure of different laminæ, and seem to open up a new field in physical optics. He is at present examining various specimens, in order to give a greater degree of generality to the results previous to laying them before the public.

---

*An Account of the Explosion of Inflammable Air which lately occurred in the Collingwood Main Colliery.*

On Saturday the 17th of July, at two o'clock, p. m. in the Collingwood Main Colliery, situated upon the river Tyne, near North Shields, a very considerable quantity of inflammable air, or carbureted hydrogen gas, came into contact with the pitmen's candles, which caused a most tremendous explosion, by which eight persons were killed upon the spot, and two severely wounded and scorched. The following particulars of this melancholy disaster were communicated verbatim at the above-mentioned colliery, a few days after the accident, to the writer of this, by Henry Hall, who fortunately escaped, though in the midst of imminent danger.

At the time when the explosion took place, the above-named Henry Hall, and five other pitmen, were proceeding with burthens of timber through the old workings or excavations (the proper road being obstructed by a creep,*) in the full confidence of safety, having been assured by Mr. Hope, the under viewer, that there was no fear of the "mine firing." In an instant this young man, Henry Hall, and the five pitmen who were with him, were by the explosion thrown upon their faces; and the shock was so great as to deprive him of sensation, as well as volition, till the after-blast, or after-damp, as it

* In working the coal, the pitmen leave pillars, in the form of parallelograms, for the support of the roof. If these pillars are narrow, and the floor of the mine soft or tender, they are apt to sink into the floor, and cause such an approximation as to prevent ventilation, &c. This is technically called *a creep*.

is called, gave him such excitement that he faintly recollects being urged like a ball along the floor of the mine with incredible velocity. Soon after this he was again deprived of sensation, in which state he continued for about twenty minutes, till he breathed the pure atmospheric air upon the bank, at the top of the shaft, to which place his brother had carried him, who descended into the mine as soon as he possibly could, upon hearing the explosion, at the risk of his own life, for the purpose of saving that of his brother, or of any other person whom he could find. H. Hall reports, that after he recovered sensation he felt his whole body racked with pain, the burnt places giving him no uneasiness, comparatively speaking; and that his suffering continued without intermission for two days. Bad as H. Hall's case was, the other five pitmen who were with him had not even such an escape, for four of them were instantly killed, and Ralph Stokell so dangerously bruised and burnt in several places, that his life was for some time despaired of.

At a distant part of the mine, where some other pitmen were employed in taking up metal plates, timber, &c. Mr. Hope, the under viewer, Mr. Wild, the overman, and two pitmen, were suffocated by the choak-damp, or carbonic acid gas. Mr. Wild had wandered at least a hundred yards before he met his death by suffocation.

Upon an explosion taking place in a coal-mine the choak-damp is very rapidly driven through all parts of the colliery from those places where it had accumulated, and the explosion is always followed by another commotion, of a still more dangerous nature, viz. the "back draught," as the miners term it. The back draught is that impetuous current of air which rushes most violently from all sides within the mine, like "the voice of mighty thunderings," to the spot where the explosion occurred, so as to overcome the vacuum which had been effected by means of the explosion.

The following is a list of the persons who were killed:—

Mr. William Hope, under viewer, leaving a wife and four children.

Mr. Ralph Wild, overman, a wife and four children.

James Campbell, pitman, a wife and child.

Ralph Hope, pitman.

Robert Clark, pitman.

Thomas Miller, pitman.

George Richardson, and William Richardson, pitmen. These two young men were brothers; and, having lost their parents, they had the filial goodness to support their grandmother, now in her 103d year, by their industry.

By the choak-damp a considerable number of horses were suffocated. In this melancholy list the dreadfully uncertain state of the pitmen is clearly demonstrated. Poor Mr. Hope, the under viewer, was heard to exclaim, in astonishment or despair, a moment before his dissolution, "God have mercy upon us; the pit has fired!" Besides the sufferers, there were fourteen or fifteen men in the pit, who, as if by a miracle, were saved. They had been employed in a distant part of the colliery; and after the explosion wandered on in darkness and stupefaction till by good fortune they chanced to arrive at that part of the mine where there was a sufficient proportion of atmospheric air to support respiration.—*Annals of Philosophy.*

IMPERIAL

### IMPERIAL INSTITUTE OF FRANCE.

*Account of the Transactions of the Imperial Institute of France for* 1812.

*(Continued from p. 256.)*

*Zoology, Anatomy, and Animal Physiology.*—M. le Chevalier Geoffroy-Saint-Hilaire, who has examined at various intervals the numerous family of bats, and has made us acquainted with many interesting species, proposes to give a general table of them. He has prefaced this undertaking with a dissertation on the rank which these singular animals ought to hold among the mammalia. They were long considered as intermediate between quadrupeds and birds. It is equally obvious that they hold an intermediate place between the quadrumania and carnivorous animals. Among the numerous arrangements proposed by naturalists, there are some, as that of Linnæus in his last editions, and that of Brisson, in which the bats are classed along with the quadrumania; in others, as that of Linnæus in his first editions, and that of Klein, they are placed with the small carnivorous animals, or eaters of insects, as the mole and the hedgehog. Some, as Storr and Cuvier, place them at the head of carnivorous animals, before the insect eaters just mentioned, and immediately after the quadrumania; with this difference, however, that Cuvier distinguishes them more particularly, and makes a subdivision of them. Others, as Ray, Blumenbach, Lacepede, and Iliger, constitute them a separate order; and this order is placed by Ray and by Lacepede in some measure out of the arrangement: by Blumenbach between the quadrumania and the other inguicula, at the head of which this naturalist places the *rongeurs*. Finally, M. Iliger places them before the carnivorous animals, at the head of which are placed, as in the arrangement of Cuvier, the devourers of insects.

It is easy to see that all these combinations will depend upon those organs to which each naturalist has paid the greatest attention. Those who have chiefly attended to the skeleton, to the intestines, to the organization of the feet, to the form of the nails, to the grinders, have considered the bats as analogous to carnivorous animals (and this is the opinion at present most followed); while those who have attended only to the fore-teeth, to the position of the mammæ, to the hanging penis, have considered them as analogous to the quadrumania.

M. Geoffroy, in the work of which we have spoken, insists more than usual upon these last relations, to which he thinks sufficient attention has not been paid. He shows particularly that the singular elongation of the anterior extremities, the general tendency of the skin to become excessively wide, and the peculiar properties which are the consequence of this in the bats, both with respect to their sensations and motions, require us to place these mammalia in a separate order; while, at the same time, their striking resemblance to the quadrumania, and to the carnivorous animals, requires that this order should be placed between them. We may look with interest to the subdivision of this order, and to the history of the species, which M. Geoffroy has promised.

M. de

M. de la Mark, employed at the Museum of Natural History in teaching every thing which concerns the animals destitute of vertebræ, published, some years ago, the work which serves as a basis to his course. He explains in it, according to his own method, the classes, orders, and genera, of these numerous animals: but as travellers have since discovered many genera and species, as anatomists have more completely explained the structure, and as the meditations of la Mark on the subject have made him discover various new relations among these animals, he has published an abridged table of his course, after his method in its most perfect state, in which he satisfies himself with giving the characters of the greater divisions, and simply enumerating the names of the genera.

He follows in their arrangement the degrees of complicateness, beginning with the most simple animals. Supposing that those which have no visible nerves only move in consequence of their irritability, he calls them *apathic animals*. He gives the names of *sensible animals* to the other animals without vertebræ, and of *intelligent animals* to those which have vertebræ. To his old classes, now well known to naturalists, he adds the *cirrhipeda*, which include the *glands-de-mer*, and other analogous animals, and which he places between his annelides and his mollusca; that of the *epizoaines*, or intestinal worms, which he places among his apathic animals; and the *infusoria*, or microscopic animals without visible mouth or intestines. He leaves the *echinodermes* in his *radiaires*, and among the apathic animals, with a greater degree of simplicity than the intestinal worms. We regret that we have not room to notice the other changes introduced by M. de la Mark into his orders, nor the numerous additions which he has made to the list of genera; but naturalists will not fail to look for them in the work itself.

Notwithstanding the success of the anatomical investigations of animals without vertebræ for several years back, there still remained a family in which the fundamental organs were not well known. It is the family called *echinodermes*, which comprehends the star-fish, and other analogous genera. The Class having proposed a prize for the perfecting of this branch of comparative anatomy, it was gained by M. Tiedeman, Professor in the University of Landshut. The memoir of this skilful anatomist makes us accurately acquainted, for the first time, with many particulars respecting the organization of these singular animals. A species of circulation is easily observed between their organs of digestion and those of respiration, without, however, offering a complete double circle. Nor can the branches be followed in the exterior organs, nor in those of motion. It appears even, according to M. Tiedeman, that a quite different vascular system is distributed to those numerous peduncles which in these animals serve for instruments of locomotion.

The organs of respiration differ much in different genera. In the holothuria they represent hollow trees, whose branches fill and empty themselves with water from without, and are interlaced with a vascular net. In the *stars* and urchins the water penetrates immediately into the cavity of the body, and moistens all the parts of it.

This

This beautiful work, accompanied by plates exquisitely finished by M. Münz, Doctor of Medicine, appeared to the Class to deserve the prize, by the number of new facts well authenticated which it presents, and by the great progress which it has made to the intimate knowledge of the echinodermes, though it has not completely answered the question proposed relative to their circulation.

A family much more simple in its organization than the echinodermes, but much more numerous in species, namely, the *corals*, and other animals composed of a solid basis, has been particularly studied by M. Lamouroux, both with respect to the species and the methodical arrangement. This naturalist has made a great collection of those whose basis is not stony, and which present forms so agreeable, and often so singular; and comparing with much care the form, the mutual position, of the cells from which the polypi issue, and all the other visible differences of these animals, he proposes to add twenty-eight new genera. This is an important work for completing the system of animals; but it does not, from its nature, admit of an abridged analysis. We are anxious for its speedy publication.

M. Cuvier, proposing soon to begin printing the great book on comparative anatomy with which he has been occupied for so many years, has presented to the Class a table of the divisions according to which the animal kingdom will be distributed in that work. Naturalists have been long struck with the great differences which separate animals without vertebræ from each other, while animals with vertebræ resemble each other in so many respects. Hence a great difficulty in generalizing that branch of comparative anatomy, while it is easy to generalize what relates to the animals with vertebræ. But this difficulty has suggested its own remedy. From the manner in which the propositions relative to each organ are always grouped, M. Cuvier concludes that there exist among animals four principal forms. The first is that which is known under the name of animals with vertebræ; and the three others are nearly similar to it in the uniformity of their respective plans. The author calls them *mollusca, articulated animals,* and *radiated animals,* or *zoophites.* He subdivides each of these forms or branches into four classes, from motives nearly similar to those which have produced the four classes generally adopted among the animals with vertebræ. He has drawn from this disposition, in some respects symmetrical, a great facility in reducing under general rules the differences of organization.

The comparison which the same author has made of the osteology of the animals with vertebræ, has given him ideas respecting the bony structure of the heads in this class, which he has likewise presented to the Class.

It had been for some time observed that the oviparous animals with vertebræ, that is to say, birds, reptiles, and fishes, had certain common relations in their structure which distinguished them from the mammalia. M. Geoffroy-Saint-Hilaire had even presented some years ago an elaborate essay on the subject, of which an account was formerly given, in which, among other things, he had shown the identity of the structure of the heads of oviparous animals, and the resemblance in the numerous pieces which enter into their composition, with that of those which we distinguish in the fœtus of mammalia, where, as is known, the bones are much more subdivided than in adults.

M. Cuvier, adopting the views of M. Geoffroy, has tried to determine in an accurate manner to what bone of the head of mammalia corresponds each group of bones in the head of the different oviparous animals; and he conceives he has succeeded, by joining to the analogy of the fœtus of the first the consideration of the position and of the functions of the bones; that is to say, by examining what organs they protect, to what nerves and vessels they give passage, and to what muscles they furnish attachments.

M. Jacobsen, Surgeon-Major in the armies of the King of Denmark, has made known to the Class an organ which he discovered in the nostrils of quadrupeds, with which no anatomist seems to have been acquainted. It consists in a narrow sack placed along the canal of the nose, defended by a cartilaginous production, covered internally by a mucous membrane, doubled in part by a glandular tissue, receiving remarkable nerves, with distinct divisions of the first pair, and which open most commonly into the palate, behind the fore-teeth, by a canal which passes through the hole called *incisive* by anatomists. This organ does not exist in man, and is more distinct in most herbivorous animals than in the carnivorous. We must suppose that it is connected with some of those faculties which nature has given to quadrupeds, but denied to our species, as that of rejecting poisonous substances, of distinguishing the sex, the state of heat, &c.

The particular history of animals is enriched with important works and interesting observations.

M. de Humboldt, Foreign Associate, has published the first volume of his *Observations on the Animals of America*, in which he has inserted not only his different researches on the condor, the electric eel, the crocodile, and many other objects of which we have spoken in our preceding analyses; but he has likewise given several new memoirs, namely, on the apes of the New World, of which Buffon and Gmelin only made known 11 or 12 species, but which Humboldt, uniting his observations with those of Azzara and Geoffroy-Saint-Hilaire, makes 46. He has recently read to the Class another memoir intended for his second volume, in which he describes new species of serpents that he found in Guyana.

The tempests which agitated the sea last winter threw ashore several large cetaceous fish upon our coasts. The Class appointed, as a commission to examine the facts which were received respecting these animals, MM. le Comte Lacepede, Geoffroy-Saint-Hilaire, and Cuvier.

These naturalists have observed that several of these animals were formerly unknown, and that this subject, which might be interesting to our fisheries and our commerce, deserved to draw the attention of government. They gave a description of a species thrown ashore in great great numbers near St. Brieux. M. Lemaout, naturalist and apothecary in that city, having collected with much care all the essential parts, it was easy to recognise a species of dolphin hitherto unknown to naturalists, and of which there only existed a bad figure in the treatise on fishes by Duhamel. It is distinguished by the globular form of the head, almost similar to an ancient helmet. Its length is about 20 feet.

We noticed last year the researches of M. Lamouroux on the innumerable and very small eels, known at the mouths of some of our rivers by the name of *montée*, and we announced the probability that they might belong to some of the little known species of this genus. M. Lamouroux has determined by new experiments that the montée is the fry of the *pimperneau*, a species of eel noticed by Lacepede in his history of fishes, and which is distinguished from the others by its pectoral fins being hollowed out like the wings of bats.

Our associate M. de la Billardiere, who employs himself in bringing up bees, having observed one whose abdomen was larger than usual, found in it a white worm, which M. Bosc examined. The body of this worm was white, divided into twelve rings, flattened below, terminated at one extremity by two large tubercles, pierced each with an oval hole, and at the other by two soft points. Under the tubercles is a transverse slit. M. Bosc, regarding this slit as the mouth, considers the part terminated by two points as that where ought to be the anus; and ranging the animal among intestinal worms, he makes a new genus of it, under the name of *dipodium*. He admits, however, that it is possible that the organs may in fact be reversed, and then the worm would much resemble the larvæ of flies with two wings. There is reason to believe, from the observations of Latreille, that the larva of one of these flies, (the *canops ferruginosa*) lives in the interior of the drones. It is very remarkable that so large a worm should inhabit the body of an insect so small as a bee.

The portion of digestion which takes place in the stomach must have early attracted the attention of physiologists, and recourse was had successively to all the powers of nature to explain it. It was long ascribed to the muscular trituration of the stomach; but Reamur having remarked that food contained in incompressible tubes, open at the two ends, was digested like other food, the general opinion since that time has been that the food was dissolved by means of a juice secreted by the stomach.

---

The Committee who undertook to make enquiry into, and ascertain the extent of the Process practised by Messrs. Delahoyde and Lucett for the Relief of Persons afflicted with Insanity, and to provide the means of paying the expense of such enquiry, have published their first Report.* It consisted of the Duke of Kent, the Duke of Sussex, Lord Dundas, Earl Fitzwilliam, Lord Milton, Viscount Melville, the Duke of Bedford, the Earl of Cork, and seven

---

* Chapple, Pall-Mall, 1s.

other gentlemen. Being informed by Dr. Harness that the government receiving-house at Hoxton contained the greatest number of the most confirmed lunatics, and having procured a list of them, the Committee on the 7th of June held their meeting at the said receiving-house. They then made an arrangement with Messrs. Delahoyde and Lucett, as to terms for receiving, in the first instance, *four patients* into their care, and afterwards such further number as might be thought proper.

On the 27th of June, *John Braily*, being one of the selected list, and who appeared to the Committee to be more violent if possible than the rest, heavily chained and handcuffed, with an horse-cloth, by way of covering, about his waist, was conducted to Sion Vale. Braily, by the list, was described as coming from his Majesty's ship the Phœnix, and it appeared that he had been received at Hoxton on the 18th of September, 1809, from whence, after remaining there twelve days, he had been removed to Bethlem Hospital, and there continued a year and eleven days, when he was, on the 11th of October, 1810, returned to Hoxton as an incurable maniac, and there remained till he was removed to Sion Vale.

*William Matters* had, it appears by the list, been a marine on-board the Revenge, and was sent to Hoxton the 16th of April, 1808, and after remaining there seven days, was sent to Bethlem Hospital, where he continued one year one month and seven days, and then returned as incurable to Hoxton, and, after remaining there four years and two months, was conducted to Sion Vale, viz. on the 30th of July.

*James Cardiff*, formerly a marine on-board the Sussex, was, as appears by the list, sent to Hoxton the 22d of February, 1810; and from thence, at the end of a month and twenty days, to Bethlem, where he remained a year and ten days, and was then returned to Hoxton, and continued there two years three months and six days previous to his removal to Sion Vale, which was on the said 30th of July.

*Daniel O'Keefe* had been quarter-master-serjeant in the 47th regiment, and confined at Hoxton, from whence he was conveyed to Bethlem, and in October, 1812, discharged as incurable, as appears by a letter from the War Office, to John Ridge, esq. the agent to the regiment, dated the 14th of October, 1812; and was discharged from the service by reason of his having been reported incurably deranged in his intellect; as appears by a letter to Mr. Ridge, dated Horse Guards, 21st of November, 1812, signed Harry Calvert. O'Keefe was then put upon the out-pension list of Chelsea Hospital, by an order dispensing with his personal appearance before the board, and being under the care of his wife at Thatcham, was by her consent removed to Brentford, and after being examined by their royal highnesses the Dukes of Kent and Sussex and Dr. Harness, was placed at Sion Vale under the care of Mr. Delahoyde.

At a meeting of the Committee, on the 7th of August, Dr. Harness was requested to take the trouble to inspect the state of these four patients at Sion Vale, which was accordingly done, as appears by his Report, dated the 27th of August, 1813.

"Upon the 10th of August, 1813, I visited the patients at Sion Vale,

Vale, accompanied by Dr. Weir, Naval Medical Inspector of Hospitals, when we found *Cardiff* assisting in brewing beer for the use of the family—continued in conversation with him some minutes, after which Dr. Weir, who had been in the habit of seeing him almost weekly during his confinement at Hoxton, pronounced him to be in a better state than he recollected ever to have seen him. Dr. Weir observed that he had frequently seen Cardiff employed in the service of the family at Hoxton.

" We afterwards saw *Braily* walking in the back garden, but I am sorry to observe not in the quiet composed state I had been accustomed to find him on my former visits; he being particularly talkative, complaining of his situation, and being constantly annoyed by numerous people, mostly women, relating their complaints to and of him; upon which I asked him, if he felt himself better than when at Hoxton; to which he replied that he certainly did; but that he did not know how long he might continue so, as he found his head becoming much confused, which observation occasioned Mr. Delahoyde to remark that he should again undergo the curative process in the evening. On Saturday the 21st instant, Mr. Delahoyde called on me at the Transport Office, to inform me that Braily had undergone the curative process on the evening of the 10th instant, and that he had derived considerable benefit therefrom.

" Upon the 25th, I visited Sion Vale, when I found Braily much more calm and collected than when seen by Dr. Weir and myself upon the 10th instant. Conversed and walked in the garden with him more than a quarter of an hour, during which time he conducted himself with great propriety, but occasionally expressed himself with more than usual rapidity, but in language strictly decorous.

" Upon the 10th instant saw *O'Keefe* and *Matters* working in the garden; they both appeared composed and reconciled to their situation. Dr. Weir thought *Matters* improved.

" Each of them, after being talked to a short time, reverted to his peculiar complaint, viz. O'Keefe to the loss of his hair, and Matters to his being distended by air.

" On my visit of the 25th instant, I could not discover that *O'Keefe* had made any progress to amendment.

" *Cardiff* and *Matters* I consider improved; they both bearing me to converse with them upon those subjects on which they had rested their principal complaints; and which subjects could not before have been touched upon without exciting considerable emotion.

" J. Harness."

The following is a copy of the minutes of the Committee, held at Sion Vale, the 15th of September, 1813, present the Duke of Kent, Thomas Smith, Esq. and Dr. Harness.

" The Committee now assembled, having conversed with and examined the four public patients at this place, with a view to form their judgment of them, came to the following opinion.

" Braily, certainly more calm and collected than on the 25th ultimo, or at any time when seen by Dr. Harness since his arrival at Sion Vale.

" O'Keefe, perfectly calm, and his general health improved, but recollection still much impaired.

"Cardiff, much better, but does not appear wholly free from the influence of the particular cause to which he attributes his complaint.

"Matters does not appear to labor under the least symptoms of complaint."

The process itself is reserved by them from the public; but having been detailed by Mr. TARDY, of Marchmont Stuart, in our Number for August last, we consider these cases as mere exemplications of his mode of treatment, and in that point of view they merit the notice of our readers.

---

*Philosophical Society of London.*—On Wednesday, October 6th, the anniversary of this Society was celebrated. The following gentlemen were elected officers and council for the year ensuing:—

*President.*—J. C. Lettsom, M. and LL.D. F.R.S. F.A.S. &c. &c.

*Vice-Presidents.*—J. Taunton, F.A.S., J. J. B. Beaumont, F.A.S., George Rees, M.D., J. Adams, M.D. F.L.S., J. Sowerby, F.L.S. G.S. W.S. &c., J. Hamilton, M.D.

*Treasurer and Secretary.*—T. J. Pettigrew, F.L.S.

*Curators.*—W. C. Pettigrew, J. Hare, junior.

*Registrar.*—J. Meers.

*Orator.*—Dr. George Rees.

*Council.*—W. Bullock, F.L.S., T. Bedder, J. Hare, R. Thompson, J. B. Brown, B. Clarkson, J. P. Heath, F. K. Cromwell, W. Henley, H. Patten, C. Holloway, W. Clarke, J. Collins, J. Andrewes, T. Lewis, R. Saumarez, T. Bittlestone, J. Wright.

The Anniversary Oration was delivered at half-past three o'clock, by Dr. Lettsom, which it is expected will be published.

---

*Surrey Institution.*—The following arrangements have been made for Lectures in the ensuing seasou:—Mr. J. Mason Good on the Philosophy of Physics, to commence on Friday the 5th of November, and to be continued on each succeeding Friday; Dr. Thompson on Chemistry, to commence on Tuesday, November 9, and to be continued on each succeeding Tuesday; and Mr. Bakewell on Natural and Experimental Philosophy, to commence early in January 1814.

---

The excellent Society for the Relief of Widows and Orphans of Medical Men in London and its vicinity, held its Anniversary Festival for 1813, on the 29th of October, and was numerously and most respectably attended.

The income of the Society for the last twelve months, ending the 15th of September, was as follows:

| | | | |
|---|---|---|---|
| By subscriptions of members and donations | £325 | 8 | 0 |
| From funded property . . . . . | 601 | 9 | 0 |
| | £926 | 17 | 0 |

In the course of last year, the treasurer has been enabled to purchase 2000*l.* stock, three per cent. consols; which brings the present capital of the Society to 24,400*l.* three per cent consols, producing the annual sum of 612*l.*, to which stock an increase is made every half year; it possesses also 200*l.* navy five per cents, producing annually 10*l.*; total annual dividends at present 622*l.*

The sum distributed among widows and orphans during the present year has been 357*l.* 10*s.*

The number of members belonging to the Society is 295.

LONDON

